# Reduced left precentral regional responses in patients with major depressive disorder and history of suicide attempts

**DOI:** 10.1371/journal.pone.0175249

**Published:** 2017-04-05

**Authors:** Noa Tsujii, Wakako Mikawa, Emi Tsujimoto, Toru Adachi, Atsushi Niwa, Hisae Ono, Osamu Shirakawa

**Affiliations:** 1 Department of Neuropsychiatry, Kindai University Faculty of Medicine, Osaka-sayama, Osaka, Japan; 2 Department of Psychological Science, Graduate School of Humanities, Kwansei Gakuin University, Nishinomiya, Hyogo, Japan; Chiba Daigaku, JAPAN

## Abstract

Previous neuroimaging studies have revealed frontal and temporal functional abnormalities in patients with major depressive disorder (MDD) and a history of suicidal behavior. However, it is unknown whether multi-channel near-infrared spectroscopy (NIRS) signal changes among individuals with MDD are associated with a history of suicide attempts and a diathesis for suicidal behavior (impulsivity, hopelessness, and aggression). Therefore, we aimed to explore frontotemporal hemodynamic responses in depressed patients with a history of suicide attempts using 52-channel NIRS. We recruited 30 patients with MDD and a history of suicidal behavior (suicide attempters; SAs), 38 patient controls without suicidal behavior (non-attempters; NAs), and 40 healthy controls (HCs) matched by age, gender ratio, and estimated IQ. Regional hemodynamic responses during a verbal fluency task (VFT) were monitored using NIRS. Our results showed that severities of depression, impulsivity, aggression, and hopelessness were similar between SAs and NAs. Both patient groups had significantly reduced activation compared with HCs in the bilateral frontotemporal regions. Post hoc analyses revealed that SAs exhibited a smaller hemodynamic response in the left precentral gyrus than NAs and HCs. Furthermore, the reduced response in the left inferior frontal gyrus was negatively correlated with impulsivity level and hemodynamic responses in the right middle frontal gyrus were negatively associated with hopelessness and aggression in SAs but not in NAs and HCs. Our findings suggest that MDD patients with a history of suicide attempts demonstrate patterns of VFT-induced NIRS signal changes different from those demonstrated by individuals without a history of suicidal behaviors, even in cases where clinical symptoms are similar. NIRS has a relatively high time resolution, which may help visually differentiate SAs from NAs.

## Introduction

Every year, one million individuals world over die from suicide [1]. This is a serious global health problem, but suicide is difficult to predict and prevent in majority of the people at risk. Although the pathogenesis of suicide is multifactorial, the strongest predictor of risk for a future attempt is a history of suicidal behavior and presence of a psychiatric disorder, particularly a major depressive disorder (MDD) [2, 3]. Although most patients with MDD never attempt suicide, those who do frequently exhibit a diathesis for suicidal behavior [4], a notion formalized in the diathesis–stress model of suicidal behavior [3]. According to this model, the proximal stressors leading to suicidal behavior are commonly psychiatric disorders together with acute psychosocial crises, whereas the components of the diathesis for suicidal behavior are impulsivity, aggression, and hopelessness.

*In vivo* brain imaging is a promising tool for identifying neurological correlates of the diathesis for suicidal behavior [5, 6]. A magnetic resonance imaging (MRI) study found that depressed patients at high suicide risk had a significantly thinner cortex in the left dorsolateral and ventrolateral prefrontal regions than those at a lower suicide risk [7]. A recent MRI study indicated that suicide attempters (SAs) with a past history of mood disorders have reduced activity in the left ventrolateral prefrontal cortices compared with healthy controls (HCs) and with patient controls with a past history of mood disorders but not of suicidal behavior [8]. Furthermore, functional MRI (fMRI) studies have shown that SAs can be distinguished from non-attempters (NAs) by specific frontal cortex activation patterns in response to angry and happy versus neutral faces [9] and by decreased activation in the medial prefrontal cortex while reading autobiographical accounts of recent suicide attempts compared with while reading neutral scripts [10]. Single-photon emission computed tomography (SPECT) studies have found that SAs have significant perfusion deficits in the prefrontal cortex [11] and bilateral superior frontal regions [12] compared with the control subjects and in the left frontal region compared with controls and depressed NAs [13]. In addition, a positron emission tomography (PET) study of patients with MDD revealed prefrontal hypofunction that was exacerbated by low serotonergic activity and was proportional to the lethality of past suicide attempts [14]. One recent investigation found that compared with depressed individuals with only suicidal thoughts, depressed individuals without suicide thoughts and plans, and healthy controls, depressed individuals with suicide plans showed relative hypometabolism in the right middle frontal gyrus and right inferior parietal lobe [15]. Thus, these neuroimaging studies have revealed altered brain structure and regional activity associated with vulnerability for suicide risk in psychiatric disorders [16, 17].

Multichannel near-infrared spectroscopy (NIRS) is a non-invasive optical technique that allows monitoring of hemodynamic changes related to cortical neural activity by measuring relative changes in oxygenated hemoglobin (oxy-Hb) and deoxygenated hemoglobin (deoxy-Hb). NIRS has several advantages over other neuroimaging techniques for psychiatric research [18]. Near-infrared light is completely non-invasive, and therefore, there are no concerns regarding radiation exposure typically observed with other neuroimaging techniques, such as SPECT/PET. In addition, its easy applicability and high ecological validity make NIRS particularly suitable for psychiatric patients who may be afraid of tight enclosures (e.g., in MRI/PET scanners) or who exhibit motor restlessness that interferes with motion-sensitive imaging methods, such as MRI, SPECT, and PET. Furthermore, NIRS has a relatively high time resolution. Thus, it is a neuroimaging tool that can be applied clinically for assessing potential biomarkers in patients with psychiatric disorders. Many studies using NIRS have demonstrated that changes in mean oxy-Hb levels in frontotemporal regions induced by a verbal fluency task (VFT) are significantly lower in patients with MDD than in control subjects [19–23]. However, it is still unclear whether these NIRS signal changes are associated with a history of suicide attempts and a diathesis for suicidal behavior in MDD.

Here we hypothesized that patients with MDD and a history of suicidal behavior can be distinguished from patients with no such history and from non-psychiatric control subjects by measuring hemodynamic responses in frontotemporal regions using NIRS. We used phonemic verbal fluency as the cognitive task to stimulate changes in hemoglobin levels because it is easy to understand and execute for patients with psychiatric disorders who present with depressive symptoms [24], and poor performance in this task has been linked to suicidal behavior in patients with psychiatric disorders [25, 26]. In addition, we examined whether VFT-induced hemodynamic responses were associated with impulsivity, aggression, and hopelessness, which would support the validity of NIRS responses as a measure of vulnerability for suicide risk in MDD. Accordingly, the aim of this study was to explore frontotemporal hemodynamic responses in depressed patients with a history of suicide attempts using 52-channel NIRS.

## Materials and methods

### Participants

The study included 68 patients (including 44 females, comprising 64.7% of study patients) with MDD and 40 HCs matched by age, gender ratio, and estimated IQ. The patients were subdivided into two groups: 1) SAs (n = 30), with a documented history of suicidal behavior and 2) NAs (n = 38), with no known history of suicidal behavior and no family history of suicide attempts among first- or second-degree family members. Suicidal behavior was defined as any act performed with the intent to die [3]. The diagnosis of MDD was made according to the criteria of the Diagnostic and Statistical Manual of Mental Disorders, 4th edition, using the Mini International Neuropsychiatric Interview (MINI; Japanese version 5.0.0) [27]. The methods used by SAs were confirmed by clinical records and interviews and were as follows: jumping from heights (n = 2), hanging (n = 7), traffic accident (n = 1), neck cutting (n = 1), wrist cutting (n = 8), and drug overdose (n = 11). HCs were screened using MINI, and candidates with a history of psychiatric disorders or heritable neurological diseases among first- or second-degree family members were excluded. Exclusion criteria included the following: 1) a history of head trauma with loss of consciousness, 2) current or previous neurological diseases, 3) current or previous endocrine diseases, 4) alcohol/substance abuse or addiction within the past 12 months, 5) a history of electroconvulsive therapy, and 6) left-handedness [28]. After they were given a complete description of the study, written informed consent was obtained from all subjects. A series of questions was used to assess the person’s understanding of key issues, e.g., the purpose of the research, the foreseeable risks, and anticipated benefits of study participation. This study complied with the Declaration of Helsinki and was approved by the Ethics Committee of the Kindai University Faculty of Medicine (No. 20–12).

### Assessment of symptoms

Depression severity was evaluated using the 17-item Hamilton depression rating scale (HAM-D) administered using a structured interview guide [29]. IQ was estimated using the Japanese version of the National Adult Reading Test [30]. Daily doses of all antidepressants were converted to an equivalent dose of imipramine; those of antipsychotics, to that of chlorpromazine; and those of anxiolytics/hypnotics, to that of diazepam [31]. Impulsivity, aggression, and hopelessness were assessed using the Barratt Impulsiveness Scale 11th [32], Buss-Perry Aggression Questionnaire [33], and Beck Hopelessness Scale [34], respectively.

### NIRS

**Fig 1:**
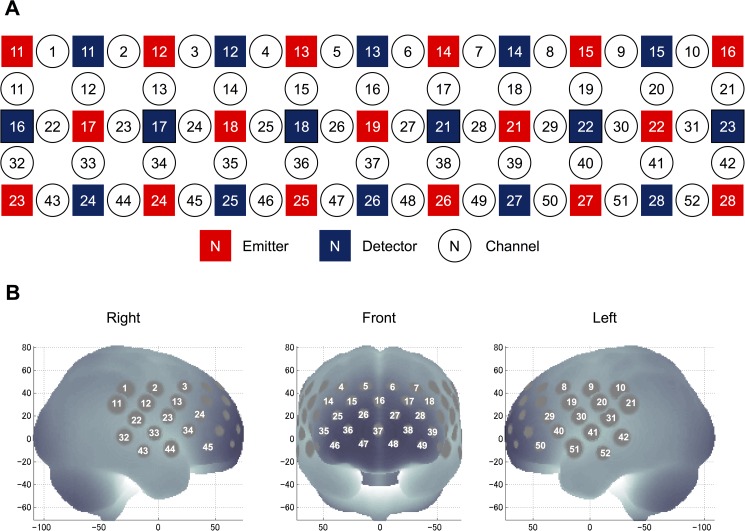
Locations of the NIRS channels. (A) Arrangement of the 17 emitters and 16 detectors and definition of the channels. (B) The anatomical site corresponding to each channel.

We used a 52-channel NIRS device (ETG-4000 Optical Topography System; Hitachi Medical Co., Tokyo, Japan) to estimate changes in regional cortical Hb concentration during the cognitive activation task, as described previously [35]. The probes (17 emitters and 16 detectors, alternating) were fixed using 3 × 11 thermoplastic shells with an inter-optode distance of 3.0 cm. Each adjoining pair of an emitter and detector was referred to as a “channel,” resulting in 52 channels in total (**Fig 1A**). The lowermost probes were positioned along the Fp1–Fp2 line according to the International 10–20 system (**Fig 1B**). The probes can measure Hb values bilaterally from the prefrontal and temporal surface regions at a depth of 20–30 mm from the scalp. This depth range corresponds roughly to the surface of the cerebral cortex.

NIRS measures relative changes in oxy- and deoxy-Hb concentrations (in mM) using two wavelengths (695 and 830 nm) of near-infrared light based on the modified Beer–Lambert law [36]. However, NIRS cannot measure the absolute path length from the emitter to detector. We, therefore, recorded relative mean changes in Hb concentration from the baseline (in mM∙mm). NIRS signals were acquired with a time resolution of 0.1 s. Mean changes in task-related oxy/deoxy-Hb levels were calculated by a linear fit to two baseline periods, the final 10 s of the pre-task period and the final 5 s of the post-task period (integral mode). We set the moving average window to 5 s to remove high-frequency noise, such as from heartbeats and small movements. Data from channels were excluded due to excessive level of artifacts using a computer algorithm adopted in previous studies [37]; thus, the number of available channels varied between individuals, but the mean number of channels did not differ between the groups [SAs: mean ± SD, 49.3 ± 3.3; NAs: mean ± SD, 48.9 ± 4.6; and HCs: mean ± SD, 49.9 ± 3.5; Kruskal–Wallis non-parametric one-way analysis of variance (ANOVA), χ^2^ = 0.19, *p* = 0.91].

The spatial information for each channel was estimated using data from the Functional Brain Science Laboratory at the Jichi Medical University, Japan [38, 39]. According to the LONI Probabilistic Brain Atlas (LPBA40) [40], NIRS channels can record functional hemodynamics within the bilateral frontal, temporal, and parietal cortices. We anatomically labeled NIRS channels only after determining the LPBA40 region of highest probability.

For the analysis of NIRS data, we focused on oxy-Hb because it is thought that cortical activation is more directly reflected by a task-related change in this parameter than by a change in deoxy-Hb, as indicated by a stronger correlation with blood oxygenation level-dependent signals measured on fMRI [41].

### Activation task

**Fig 2:**
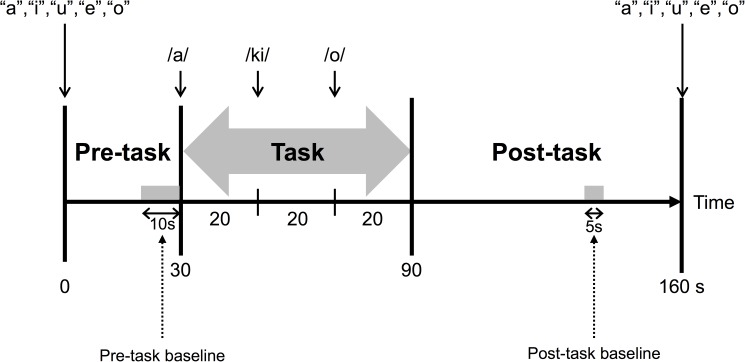
Design of the verbal fluency task.

Changes in hemoglobin levels were stimulated using VFT because previous studies have shown measurable prefrontal activation during VFT in healthy subjects [19, 42]. The task procedure was similar to that described previously [35]. VFT used in the present study included a 30-s pre-task baseline period, 60-s task period comprising three 20-s blocks, and 70-s post-task baseline period. During the pre- and post-task baseline periods, the subjects were instructed to repeat a train of syllables (“a, i, u, e, and o”). During the 60-s task, subjects were asked to generate as many words as possible starting with that syllable (**Fig 2**). The possible syllables were as follows: block 1 (0–20 seconds), “a,” “to,” or “na”; block 2 (20–40 seconds), “i,” “ki,” or “se”; and block 3 (40–60 seconds), “o,” “ta,” or “ha.” The total the number of correct words represented the subject’s performance score.

### Statistical analysis

The threshold for statistical significance was set at *p* < 0.05 (two-tailed). Demographic and clinical variables were compared between the study groups using a χ^2^ test for categorical variables and t-test, Mann–Whitney U tests, or ANOVA (with Tukey’s post hoc test) for continuous variables.

To identify regional differences in VFT-induced frontotemporal hemodynamic responses, mean oxy-Hb changes among the study groups were compared using an ANOVA with a Bonferroni-corrected threshold of *p* = 0.00096 (i.e., 0.05/52), followed by Tukey’s post hoc test with a threshold of *p* = 0.05. To examine the relationships between mean oxy-Hb changes and demographic and clinical variables, we calculated Pearson’s correlation coefficients, again with the Bonferroni-corrected threshold of *p* = 0.00096.

All statistical tests were performed using IBM SPSS Statistics for Mac (version 22.0; IBM Corporation, Armonk, NY, USA).

## Results

### Demographic and clinical characteristics

**Table 1** summarizes the demographic and clinical characteristics of the study groups. There were no significant differences between the three groups in any of the demographic variables. As expected, both MDD patient groups scored higher than HCs on impulsivity, aggression, and hopelessness, whereas no significant differences were observed between the SA and NA groups. In addition, both MDD patient groups showed poorer performance than HCs on VFT. Thus, SAs and NAs had similar clinical symptoms.

**Table 1:** Demographic, clinical, and neuropsychological characteristics of suicide attempters, non-attempters, and healthy controls.

	Patients with MDD		HCs (*n* = 40)	Analysis	
	SAs (*n* = 30)	NAs (*n* = 38)		Statistic	*p* value
Demographic characteristics					
Age (years)	37.6 ± 10.0	38.8 ± 9.7	38.2 ± 10.5	F _(df = 2,105)_ = 0.13	0.88
Females (*n*, %)	22 (73.3)	22 (57.9)	25 (62.5)	χ^2^ = 1.76	0.41
Estimated IQ	104.5 ± 9.0	102.1 ± 8.6	105.9 ± 9.7	F _(df = 2,105)_ = 1.72	0.18
Duration of illness (years)	10.2 ± 6.3	8.8 ± 7.8		U = 457.0	0.16
Comorbid axis 1 disorder (*n*, %) ^a^	14 (46.7)	13 (34.2)		χ^2^ = 1.01	0.30
Clinical variables					
Depression severity	14.9 ± 4.0	16.0 ± 4.6		U = 507.5	0.44
Impulsivity	101.5 ± 19.9	97.6 ± 19.4	81.8 ± 10.0	F _(df = 2,105)_ = 14.4	<0.001^b^
Aggression	66.0 ± 14.6	61.4 ± 9.9	51.6 ± 9.8	F _(df = 2,105)_ = 15.0	<0.001 ^b^
Hopelessness	14.3 ± 4.4	13.6 ± 3.9	4.2 ± 2.8	F _(df = 2,105)_ = 88.6	<0.001 ^b^
Verbal fluency task performance	12.9 ± 5.5	13.2 ± 4.4	16.3 ± 5.2	F _(df = 2,105)_ = 5.06	0.008 ^c^
Antidepressant doses^d^	90.8 ± 91.9	83.1 ± 91.9		U = 532.5	0.64
Antipsychotic doses^e^	19.6 ± 51.7	50.4 ± 108.9		U = 565.5	0.95
Benzodiazepine doses^f^	12.1 ± 17.2	8.3 ± 12.1		U = 519.0	0.51

Abbreviations: HCs, healthy controls; MDD, major depressive disorder; NAs, non-attempters; SAs, suicide attempters.

^a^Comorbid anxiety axis 1 disorder included panic disorder, agoraphobia, social phobia, obsessive-compulsive disorder, post-traumatic stress disorder, generalized anxiety disorder, and eating disorder.

^b^SAs and NAs had higher scores than HCs, but no significant difference was observed between SAs and NAs.

^c^SAs and NAs had lower scores than HCs, but no significant difference was observed between SAs and NAs.

^d^Antipsychotic dosages were evaluated using chlorpromazine equivalent dosage.

^e^Antidepressant dosages were evaluated using imipramine equivalent dosage.

^f^Anxiolytic dosages were evaluated using diazepam equivalent dosage.

### NIRS data

**Fig 3:**
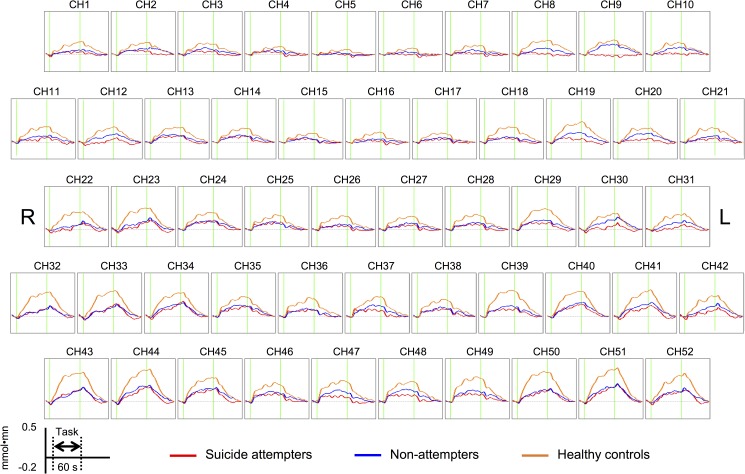
The grand-averaged waveforms of changes in the oxy-Hb signal in suicide attempters, non-attempters, and healthy controls.

**Fig 3** shows the grand-averaged waveforms of changes in the oxy-Hb signal in the SA, NA, and HC groups.

#### Within-group differences

The HC group showed significantly increased mean oxy-Hb levels from the pre-task baseline to the VFT period in 49 channels (ch1–3 and ch7–52, *t* = 3.98–9.49, Bonferroni-corrected *p* < 0.00096). The NA group showed significant activations in 29 channels (ch9–11, ch13–14, ch19–20, ch24–25, ch28–30, ch34–41, and ch44–52; *t* = 3.60–7.06, Bonferroni-corrected *p* < 0.00096). In contrast, the SA group showed significant activations in 9 channels (ch13, ch24, ch29, ch35, ch38–39, ch45, and ch48–49; *t* = 3.72–5.46, Bonferroni-corrected *p* < 0.00096). Thus, VFT induced widespread frontotemporal cortical activation in HCs and NAs, whereas SAs showed significant activation only in the left superior frontal gyrus (ch48), left middle frontal gyrus (ch38 and ch49), bilateral inferior frontal gyrus (right: ch24, ch35, and ch45; left: ch29 and ch39), and right precentral gyrus (ch13).

#### Between-group differences

**Table 2** shows a comparison between the groups of VFT-induced regional hemodynamic responses, with a Bonferroni-corrected significance threshold of *p* < 0.00096 applied.

**Table 2:** Comparison of regional hemodynamic responses of suicide attempters, non-attempters, and healthy control subjects threshold of p < 0.00096.

			MINI coordinates			Mean oxy-Hb changes				
Estimated area	R/L	NIRS channel	x	y	z	SAs	NAs	HCs	F value	Post hoc
Middle frontal gyrus	L	8	−46	23	44	0.013 ± 0.068	0.039 ± 0.077	0.113 ± 0.118	11.2	SA < HC, NA < HC
	L	18	−42	42	32	0.038 ± 0.072	0.038 ± 0.067	0.125 ± 0.111	12.3	SA < HC, NA < HC
	L	19	−55	18	31	0.035 ± 0.067	0.072 ± 0.066	0.160 ± 0.113	19.3	SA < HC, NA < HC
	L	28	−35	58	20	0.046 ± 0.072	0.053 ± 0.081	0.126 ± 0.127	7.5	SA < HC, NA < HC
	L	49	−35	63	−4	0.072 ± 0.071	0.097 ± 0.109	0.194 ± 0.143	11.4	SA < HC, NA < HC
Inferior frontal gyrus	R	34	59	27	8	0.065 ± 0.096	0.092 ± 0.104	0.198 ± 0.128	13.5	SA < HC, NA < HC
	R	45	53	43	−6	0.093 ± 0.100	0.108 ± 0.094	0.194 ± 0.148	7.7	SA < HC, NA < HC
	L	29	−52	36	19	0.065 ± 0.083	0.076 ± 0.070	0.172 ± 0.121	13.7	SA < HC, NA < HC
	L	39	−44	53	6	0.064 ± 0.075	0.084 ± 0.086	0.217 ± 0.144	21.2	SA < HC, NA < HC
	L	40	−57	28	7	0.076 ± 0.113	0.091 ± 0.096	0.202 ± 0.164	9.2	SA < HC, NA < HC
	L	50	−51	45	−6	0.066 ± 0.132	0.088 ± 0.094	0.247 ± 0.174	18.0	SA < HC, NA < HC
Precentral gyrus	R	23	64	8	20	0.025 ± 0.120	0.049 ± 0.109	0.171 ± 0.136	12.2	SA < HC, NA < HC
	L	9	−57	−1	43	−0.011 ± 0.094	0.073 ± 0.087	0.107 ± 0.116	11.4	SA < NA, SA < HC
	L	30	−62	10	20	0.008 ± 0.101	0.063 ± 0.073	0.119 ± 0.139	7.8	SA < HC
Postcentral gyrus	R	12	66	−10	31	−0.004 ± 0.070	0.047 ± 0.084	0.102 ± 0.112	11.1	SA < HC, NA < HC
	L	20	−64	−8	31	0.019 ± 0.072	0.063 ± 0.101	0.113 ± 0.110	7.5	SA < HC
	L	31	−67	−17	19	0.005 ± 0.087	0.044 ± 0.099	0.111 ± 0.137	7.8	SA < HC, NA < HC
Middle temporal gyrus	R	32	71	−29	2	0.035 ± 0.094	0.040 ± 0.098	0.176 ± 0.189	12.1	SA < HC, NA < HC
	R	43	69	−13	−10	0.070 ± 0.139	0.61 ± 0.107	0.234 ± 0.193	14.5	SA < HC, NA < HC
Superior temporal gyrus	R	22	68	−19	18	0.035 ± 0.094	0.040 ± 0.098	0.176 ± 0.189	12.3	SA < HC, NA < HC
	R	33	66	−4	5	0.056 ± 0.126	0.049 ± 0.112	0.192 ± 0.178	10.8	SA < HC, NA < HC
	R	44	60	11	−8	0.075 ± 0.127	0.109 ± 0.104	0.249 ± 0.193	13.4	SA < HC, NA < HC
	L	51	−57	14	−8	0.074 ± 0.177	0.111 ± 0.139	0.239 ± 0.216	7.6	SA < HC, NA < HC

Abbreviations: HCs, healthy controls; MDD, major depressive disorder; NAs, non-attempters; SAs, suicide attempters.

**Fig 4:**
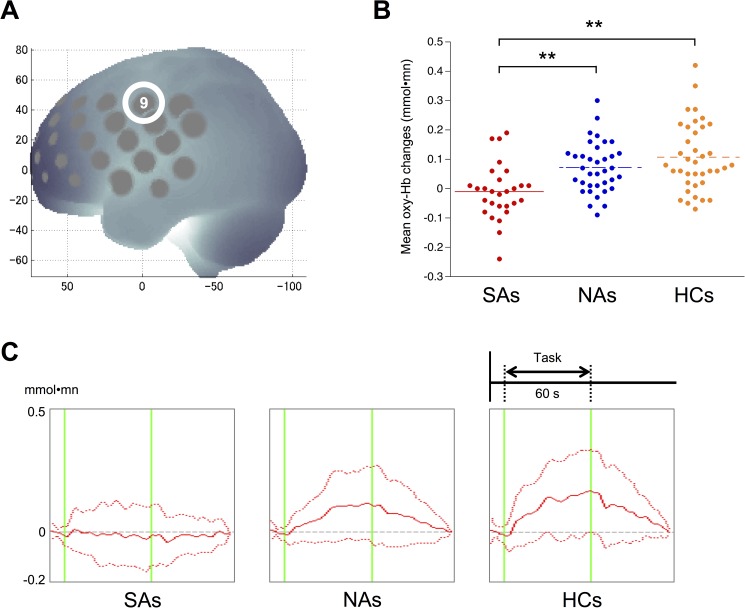
The differential time course of the oxy-Hb signal in the left precentral gyrus in suicide attempters, non-attempters, and healthy controls. (A) The anatomical site corresponding to channel 9 (left precentral gyrus). (B) Dot plots of mean oxy-Hb changes of the left precentral gyrus in SAs, NAs, and HCs. (C) The differential time course of oxy-Hb signal in SAs, NAs, and HCs in the left precentral gyrus. In NAs and HCs, the time course of changes in the oxy-Hb signal showed a gradual increase followed by a gradual decrease after the end of the task; SAs did not exhibit this response. The dashed red lines indicate standard deviations for each group. Abbreviations: HCs, healthy controls; NAs, non-attempters; SAs, suicide attempters **p < 0.01

Compared with the HC group, both MDD patient groups exhibited significantly small mean oxy-Hb changes in the left middle frontal, bilateral inferior frontal, right precentral, bilateral postcentral, right middle temporal, and bilateral superior temporal gyri. Post hoc analyses revealed that the SA group exhibited a smaller hemodynamic response than the NA (*p* = 0.0038) and HC (*p* < 0.0000) groups in the left precentral gyrus (ch9; **Fig 4A** and **4B**), whereas no difference was observed between the NA and HC groups (*p* = 0.31). In this region, patterns in the mean oxy-Hb signals differentiated the SA group from the NA and HC groups (**Fig 4C**): the time course of changes in the oxy-Hb signal gradually increased during the task and gradually decreased after it ended in the NAs and HCs, whereas SAs did not show this response.

#### Correlation between NIRS data and symptoms

**Fig 5:**
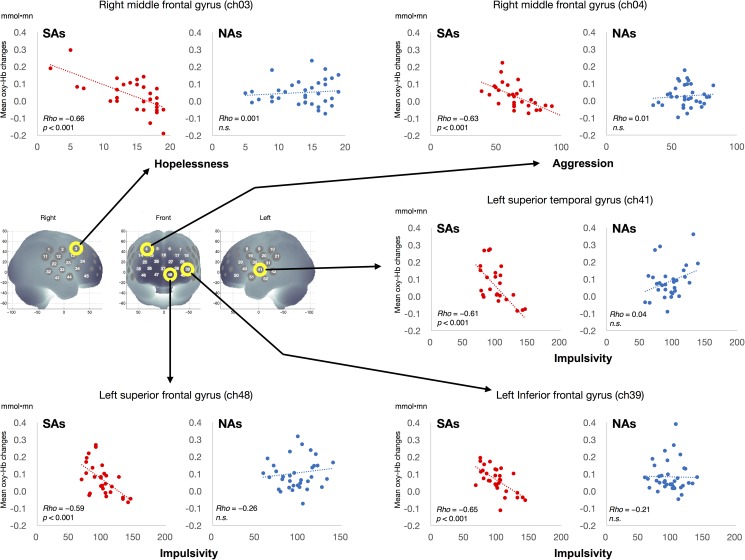
Correlation between hemodynamic responses and impulsivity, aggression, and hopelessness in suicide attempters and non-attempters. The reduced response in the left inferior frontal gyrus was negatively correlated with impulsivity level and the responses in the right middle frontal gyrus were negatively associated with hopelessness and aggression in suicide attempters (SAs) but not in non-attempters (NAs).

Significant negative correlations were found in SA group between impulsivity and the hemodynamic responses induced by VFT in the left inferior frontal gyrus (ch39; *r* = −0.64, *p* = 0.00013; **Fig 5**), the left superior frontal gyrus (ch48; *r* = −0.59, *p* = 0.00078), and the left superior temporal gyrus (ch41; *r* = −0.61, *p* = 0.00095). In addition, in this group there was a significant negative correlation between aggression and the hemodynamic response in the right middle frontal gyrus (ch4; *r* = −0.63, *p* = 0.00017) and between hopelessness and the response in the right middle frontal gyrus (ch3; *r* = −0.66, *p* = 0.00007). In contrast, the NA group showed no statistically significant correlations between any demographic or clinical variable and the hemodynamic responses.

Furthermore, we did not find any significant correlations between the hemodynamic responses and doses of antidepressants in either MDD patient groups. In addition, only the HC group showed a significant positive correlation between VFT task performance and the hemodynamic responses in the left superior temporal gyrus (ch41; *r* = 0.57, *p* = 0.00015).

## Discussion

This, to the best of our knowledge, is the first NIRS study to explore the distinct functional abnormalities of the left precentral gyrus in patients with MDD with a history of suicidal behavior. We observed a reduction in the VFT-induced hemodynamic responses in the bilateral frontotemporal regions in both MDD patient groups relative to the responses of HCs, with SAs displaying smaller hemodynamic responses in the left precentral gyrus than NAs and HCs. In this region, SAs exhibited virtually no activation from the pre-task baseline through the VFT period, in contrast to HCs and NAs. Our findings suggest that MDD patients with a history of suicide attempts demonstrate patterns of NIRS signal changes in the left precentral gyrus different from those demonstrated by individuals without a history of suicidal behaviors, even in cases where clinical symptoms are similar. NIRS has a relatively high time resolution, which may help visually differentiate SAs from NAs.

Recently, several neuroimaging studies have suggested the importance of the left precentral gyrus in the neuropathological processes underlying MDD. Left precentral gyrus thickening has been observed in healthy individuals with negative cognitive styles [43] and in patients with MDD [44]. One prospective longitudinal study has reported that progressive cortical thinning in the left precentral gyrus is associated with an increased risk for mood disorders [45]. The precentral gyrus may be of particular relevance for the etiology of suicide because of its potential role in impulsivity regulation. This region is involved in the executive functioning of response inhibition [46, 47] and is associated with other motor areas, such as the pre-supplementary motor area, which is crucial for inhibitory control [48–50]. Deficits in inhibitory control are associated with a greater propensity to act on suicidal or aggressive feelings [3]. This suggests that an important threshold for conversion to overt suicidal behavior may be functional abnormalities in the precentral region associated with impulsivity control. Furthermore, previous studies support the possibility that precentral cortical abnormalities are linked to vulnerability for suicide risk in patients with MDD [51, 52]. These lines of evidence support our results that NAs show the lack of an abnormal response. Our study suggests that the deficit in hemodynamic response measured using NIRS in the left precentral gyrus may play a distinct role in the vulnerability for suicide risk in patients with MDD.

We have reported a peculiar dysfunction of the left precentral gyrus in SAs; however, the choice of VFT as the cognitive task is debatable because thus far, a relationship between verbal fluency and suicidal behavior has not been confirmed. Recent NIRS studies have reported that social adaptation level, which might influence the risk of suicidal behavior [53], is associated with the dorsolateral prefrontal function [54], and the bilateral ventrolateral prefrontal function, and the anterior part of the temporal function [55]. However, the validity of NIRS as a measure of the vulnerability for suicide risk is supported by the associations that we observed between VFT-induced hemodynamic responses and diathesis for suicidal behavior (impulsivity, aggression, and hopelessness). We found significant negative correlations between aggression and hopelessness levels and mean oxy-Hb change in the right middle frontal gyrus. This region includes the dorsolateral prefrontal area, which also plays a critical role in the development of suicidal behavior [16, 17]. A previous investigation using NIRS for MDD also reported that the severity of suicidal ideation was associated with hemodynamic responses in dorsolateral prefrontal region [23]. In our study, SAs showed significant correlation between the severity of suicide ideation and hemodynamic responses in the right dorsolateral prefrontal region (ch45; *r* = −0.39, *p* = 0.037; uncorrected), but NAs did not show such correlation. Our results suggest that the relationship of levels of aggression and hopelessness with VFT-induced hemodynamic responses in the right dorsolateral prefrontal cortex in SAs is different from that in NAs and HCs; this region, in particular, may be related to the distinct pathophysiology of suicidal behavior in patients with MDD.

Furthermore, we demonstrated significant negative correlations between impulsivity level and hemodynamic responses in the left inferior frontal (including the dorsolateral prefrontal region), left superior frontal (including the orbitofrontal region), and left superior temporal gyri in SAs. Previous studies have reported that abnormalities in these regions are associated with suicidal behaviors in patients with MDD [11] and in those with schizophrenia [56]. Furthermore, a PET study concluded that the functional abnormality of the frontal cortex in SAs is related to the degree of impulsivity [14]. Neuroimaging studies have shown that impaired impulse control is associated with a whole-brain binding potential of the serotonin transporter in SAs [57, 58]. Thus, our results are supported by there being a regional serotonin deficiency in depressed SAs because a deficiency in serotonin input to the prefrontal cortex contributes to impulsive traits associated with a greater risk for suicidal behavior.

### Limitations

There were several methodological limitations in our study. First, we neither assessed the suicidality of the subjects using a validated instrument nor stratified the group of patients by suicidal behavior (e.g., according to violent, highly lethal, or repeated attempts). We were unable to obtain collateral verification of the history of suicidal behavior from a family member for all subjects. Second, most participants with MDD were taking psychotropic medications, even on the day of the scan. Although, thus far, there have been no reports of clear evidence of the effects of medication on NIRS signals, a recent longitudinal NIRS study reported no differences in VFT-induced oxy-Hb changes between pre- and post-antidepressant treatment time points in a MDD cohort, despite significant improvements in symptoms of depression [22]. Third, we reported that one channel (ch9) showed a significant difference between the SA, NA, and HC groups. However, our study sample size was relatively small, and it might not be able to insist on the role of difference between SAs and NAs. If the sample size is increased, the difference between NAs and HCs in the left precentral gyrus might also become significant, which means the possibility of a type II error. Fourth, although the socioeconomic background could affect our results, we did not assess the subjects’ or parental socioeconomic status using a validated instrument. Finally, we did not formally evaluate personality traits or differences between males and females, and it will be important in future studies to examine these issues.

## Conclusions

Our study demonstrated that patients with MDD and a history of suicide attempts exhibit patterns of NIRS signal changes in the left precentral gyrus that are different from those exhibited by individuals without a history of suicidal behavior and by healthy control subjects. NIRS has a relatively high time resolution, which may help visually differentiate depressed suicide attempters from non-attempters. Our results also suggest that the observed VFT-induced hemodynamic responses in frontotemporal regions are associated with the diathesis for suicidal behavior. Thus, we found unique pathophysiological features of suicidality in patients with MDD. Compared with other neuroimaging tools, the ease of applicability of NIRS may be particularly advantageous in longitudinal follow-ups that require repeated measurements to predict future suicidal behaviors in patients with MDD. Further NIRS studies of MDD are warranted. If confirmed, our findings may define a novel biomarker for suicide risk in MDD.
